# Metal Complexes of Oxadiazole Ligands: An Overview

**DOI:** 10.3390/ijms20143483

**Published:** 2019-07-16

**Authors:** Giovanni Salassa, Alessio Terenzi

**Affiliations:** 1 Department of Physical Chemistry, University of Geneva, 30 Quai Ernest-Ansermet, 1211 Geneva 4, Switzerland; 2 Donostia International Physics Center, Paseo Manuel de Lardizabal 4, 20018 Donostia, Spain

**Keywords:** oxadizole, 1,2,4-oxadizole, 1,3,4-oxadizole, metal complexes

## Abstract

Oxadizoles are heterocyclic ring systems that find application in different scientific disciplines, from medicinal chemistry to optoelectronics. Coordination with metals (especially the transition ones) proved to enhance the intrinsic characteristics of these organic ligands and many metal complexes of oxadiazoles showed attractive characteristics for different research fields. In this review, we provide a general overview on different metal complexes and polymers containing oxadiazole moieties, reporting the principal synthetic approaches adopted for their preparation and showing the variety of applications they found in the last 40 years.

## 1. Introduction

**Figure 1:**
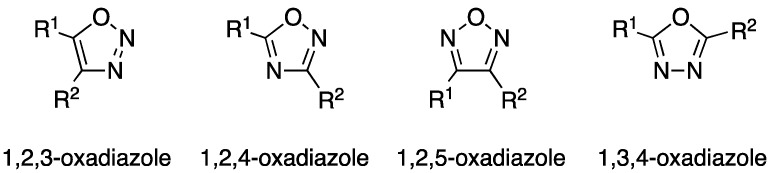
Structures of the different oxadiazole isomers.

Oxadiazoles are an interesting class of five-membered heterocyclic compounds containing two atoms of nitrogen and one atom of oxygen. They exist in four different regioisomeric forms, namely 1,2,3-, 1,2,4-, 1,2,5-, and 1,3,4-oxadiazoles (Figure 1). 1,2,4- and 1,3,4-isomers are by far more represented in literature, while the 1,2,5-isomer, which displays a different orientation of the side chains (R^1^ and R^2^), is significantly less reported [1]. 1,2,3-Oxadizole cycles are instead quite unstable and these compounds are usually observed in their diazoketone tautomeric form and are relatively complicated to synthesize [2]. It is interesting to note that both 1,2,4- and 1,3,4-oxadiazoles satisfy the Hückel rule, making them aromatic molecules. Nevertheless, 1,2,4-isomers are the least aromatic five-membered heterocyclic systems and they are better described as a conjugated dienes, while 1,3,4-oxadiazole derivatives show far greater aromaticity [2,3].

In the past 40 years, oxadiazole-based ligands stimulated the curiosity of many researchers, creating a vast literature that spans from synthesis to different applications. In fact, oxadiazoles exhibit a broad range of uses: in medicinal chemistry they were employed as drug candidates for several diseases, in organic synthesis as useful intermediates, and in material science as building blocks for new polymers. A detailed discussion on the synthetic strategies and applications concerning oxadiazoles is out of the scope of this article and different reviews covering these topics are available elsewhere [1,2,3,4,5,6,7,8]. At the same time, considering the importance of coordination chemistry in medicinal and material sciences, is somehow surprising to find out that an overview on the interaction between oxadiazole ligands and metal ions is missing, at least to the best of our knowledge. The scope of this review article is to summarize some of the most important achievements in the development of metal complexes containing oxadiazole ligands. For the ease of the reader, this contribution is divided in four main sections. In the first one, we discuss the use of oxadiazole-based scaffolds for the construction of inorganic compounds, either coordination polymers or mononuclear complexes. In the second part, we highlight those metal complexes containing oxadiazole ligands with biological activity. In the third part, we show several examples of oxadiazole-based materials that have been employed in optoelectronics. Finally, we describe how oxadiazole ligands were employed in the preparation of several metal ion sensors.

## 2. Supramolecular Assemblies and Synthetic Aspects

Notwithstanding that oxadiazole rings have been used for many years in classical organic chemistry leading to molecules with several applications, their use as scaffolds for inorganic compounds is relatively recent. The group of Miao Du, in the early 2000′s, was basically the first to establish a successful research line based on the synthesis and characterization of coordination polymers where pyridyl-substituted 1,3,4-oxadiazole were used as the main building blocks [9,10,11,12,13,14,15,16]. The idea of Du and colleagues was to coordinate 1,3,4-oxadiazole ligands to different metals in order to control the design of multidimensional infinite architectures with potential interest for new materials. Of course, prediction of a metal–organic framework topology is not trivial, but Du and collaborators worked extensively on these systems, providing a good repertoire of structures varying the oxadiazole substituents, the metal ions involved, and the corresponding counterions.

**Figure 2:**
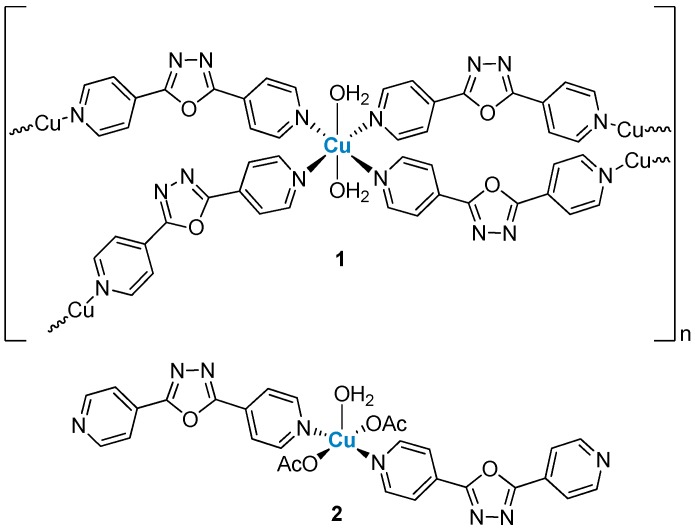
Structures of two Cu(II) complexes with the ligand 2,5-bis(4-pyridyl)-1,3,4-oxadiazole.

The first system was developed using Cu(II) and the ligand 2,5-bis(4-pyridyl)-1,3,4-oxadiazole (La), which has a 137° angle between the central oxadiazole ring and the two N-atoms of the 4-pyridines. Two compounds have been synthesized quite straightforwardly by mixing in methanol/water the ligand and Cu(II) perchlorate or acetate salts in molar ratios of 2:1, obtaining {[Cu(La)_2_(H_2_O)_2_](ClO_4_)(OH)(H_2_O)_2.5_}_n_ (**1**) and [Cu(La)_2_(OAc)_2_(H_2_O)](H_2_O)_2_(CH_3_OH) (**2**), respectively (Figure 2). The crystal structure of **1** revealed a Cu(II) diamondoid motif. The Cu(II) atom presented an octahedral geometry, characterized by a considerable Jahn–Teller distortion, with four trans oxadiazole ligands and two axial water molecules. Interestingly enough, the bent geometry of the oxadiazole scaffold afforded a structure where the metal centers acted as distorted tetrahedral nodes, generating a pair of complementary and interpenetrating diamondoid networks [9].

When the same reaction was performed using Cu(II) acetate instead of the perchlorate salt the authors did not obtain a coordination polymer but an unexpected mononuclear complex (compound **2**, Figure 2) where the Cu(II) center is coordinated to two oxadiazole ligands, two acetate anions and one H_2_O molecule at the apical position [9]. 

Considering the important role of the counterions in the final assembly, Miao Du et al. synthesized other Cu(II) complexes by applying anion-exchange procedures on **1**. After the addition of NaN_3_ and of Na_2_SO_4_ to an aqueous solution of **1**, two new compounds were obtained, {[CuLa(N_3_)_2_](H_2_O)1.5}_n_ and {[CuLa(H_2_O)(SO_4_)](H_2_O)_2_}_n_, respectively [11]. Starting from an interpenetrating diamondoid array, this process led to two different anion-dependent 3-D acentric open frameworks. The crystal structures of the two new compounds revealed linear [-CuLa-]_n_ infinite chains linked by layers of anions connecting the metal centers [11].

The same ligand was then used to synthesize metal complexes with other metal centers, including Mn(II), Fe(II), Fe(III), Co(II), and Zn(II) [14]. Relatively easy to perform reactions in H_2_O (or H_2_O/CH_3_OH and H_2_O/CH_3_CN) under reflux for 30 min of the mixture constituted by the ligand and the perchlorate salt of the selected metal (1:1 molar ratio) afforded new supramolecular complexes of the general formula [M(La)_2_(H_2_O)_4_] · (La)_2_ · (ClO_4_) ·(solvent). The authors again proved that the choice of the counterion of the metal was crucial and when they used Co(NO_3_)_2_ instead of Co(ClO_4_)_2_, a 1-D polymeric chain complex {[Co(La)(H_2_O)_2_(NO_3_)_2_](H_2_O)_3_}_n_ was obtained [14].

**Figure 3:**
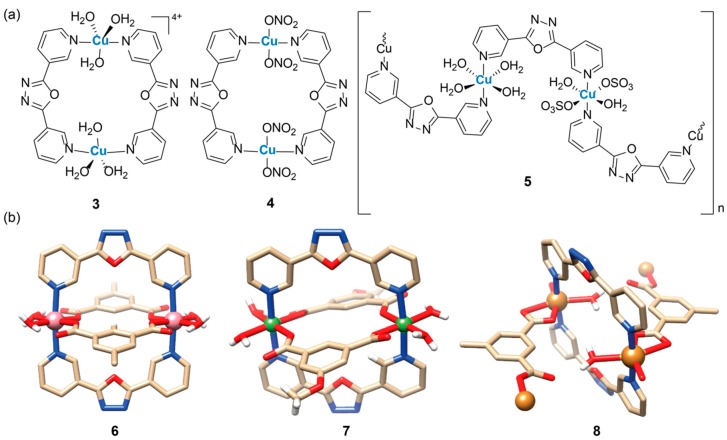
(**a**) Structures of three Cu(II) complexes with the ligand 2,5-bis(3-pyridyl)-1,3,4-oxadiazole. (**b**) Crystal structure of Co(II), Ni(II), and Cu(II) complexes with the ligands 2,5-bis(3-pyridyl)-1,3,4-oxadiazole and substituted isophthalates. Picture is adapted from reference [15] and was generated with Chimera software [9] using the original CIF data.

As a continuation of their work, Du et al. selected a slightly different oxadiazole ligand, namely 2,5-bis(3-pyridyl)-1,3,4-oxadiazole (Lb), and synthesized other Cu(II) complexes by keeping constant the solvent (CH_3_CN-H_2_O) and the metal/ligand ratio (1:1) and changing only the counterion (ClO_4_^−^, NO_3_^−^ and SO_4_^2−^) [10]. Three new compounds were obtained, [Cu_2_(Lb)_2_(H_2_O)_6_](ClO_4_)_4_(H_2_O)_4_ (**3**), [CuLb(NO_3_)_2_]_2_(CH_3_CN)_2_ (**4**), and {[Cu_2_(Lb)_2_(H_2_O)_6_(SO_4_)_2_](H_2_O)_6_}_n_ (**5**) (Figure 3a). The authors resolved the crystal structures of compounds **3** and **4** and found two dinuclear metallacyclophanes. In compound **3** the Cu(II) centers are coordinated with the pyridines of two oxadiazole ligands and three H_2_O molecules forming a distorted square-pyramid geometry. In derivative **4** the water molecules are substituted by two O donors of the nitrate counterion to form a square-planar geometry (square pyramid if two CH_3_CN molecule of the solvent are considered) [10]. On the other hand, when SO_4_^2^- was used as counterion the crystal structure of the final compound **5** showed a neutral 1-D coordination polymer, in which two independent octahedral Cu(II) centers are bridged by cisoid-I type ligands Lb (Figure 3a) [10]. 

Using a layer-separation diffusion method, the group of Miao Du prepared another six metal complexes with the oxadiazole ligand 2,5-bis(3-pyridyl)-1,3,4-oxadiazole (Lb), with 5-methylisophthalate (mip) or 5-methoxyisophthalate (moip) as ancillary ligands [15]. The synthetic procedure was the same for all compounds and consisted of dissolving the metal nitrate salt in water followed by a careful layering of a methanolic solution of Lb and mip (or moip) in a 1:1:1 molar ratio. This method facilitated the slow growth of single crystals of two Cu(II), two Ni(II), and two Co(II) compounds. The observed structural differences between these complexes can be mainly attributed to the different metal ions and dicarboxyl ligands used. Analysis of the structures showed that Co(II) and Ni(II) complexes, for example [Co(mip)(Lb)(H_2_O)_2_]_2_(CH_3_OH)_2_ (**6**) and [Ni_2_(moip)_2_(Lb)_2_(H_2_O)_4_](CH_3_OH)(H_2_O)_3_ (**7**) in Figure 3b, exhibit cage-like dinuclear coordination motifs, whereas the Cu(II) complexes display a tube-like 1-D infinite pattern, like for {[Cu(mip)(Lb)(H_2_O)](H_2_O)0.5}_n_ (**8**) [15].

**Figure 4:**
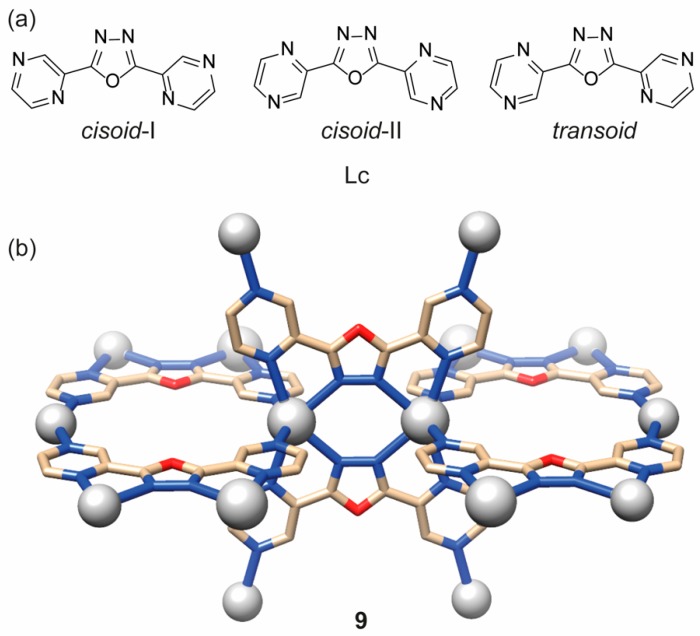
(**a**) Structures of the ligand 2,5-bis(pyrazine)-1,3,4-oxadiazole (Lc) and (**b**) its Ag(I) coordination polymer. Picture is adapted from reference [12] and was generated with Chimera software [17] using the original CIF data.

In 2005, Du et al. explored the reaction of Ag(I) salts (X= BF_4_^-^, AsF_6_^-^, CF_3_SO_3_^-^, and SbF_6_^-^) with another oxadiazole ligand, 2,5-bis(pyrazine)-1,3,4-oxadiazole (Lc). This ligand was chosen to achieve different metal–organic topologies for its higher number of potential coordination sites and different possible configurations (Figure 4a) when compared to ligands La and Lb [12]. The compounds obtained in CHCl_3_/OH(CH_2_)_2_OH/CH_3_OH media gave four coordination polymers with the general formula {[Ag(Lc)]-X·solvent}_n_. The crystal structure of all compounds showed that, in this case, the effect of the anion is limited, and the final arrangement consisted of 3-D open coordination networks of the type shown in Figure 4. In compound **9**, as an example, each silver atom is coordinated to two ligands (in the cisoid-II configuration) via chelation to a pyrazine nitrogen and an oxadiazole nitrogen and to a further two pyrazine nitrogen atoms from two different ligands. At the same time, each oxadiazole ligand is coordinated to four silver atoms leading to pseudo-tetrahedral nodes [12].

One year later, Du and co-workers accomplished new syntheses using Lc and Cd(II) or Co(II) perchlorate salts in the presence of thiocyanate [13]. These reactions, performed in CH_3_CN/H_2_O under reflux for 30 min, in a 1:1:3 (metal:ligand:SCN) stoichiometric ratio, afforded two distinct complexes: [Cd(Lc)_2_(SCN)_2_]_n_ (**10**) and a 3-D hydrogen-bonded assembly [Co(Lc)_2_(SCN)_2_(H_2_O)_2_](CH_3_CN)_2_(H_2_O)_2_ (**11**).

**Figure 5:**
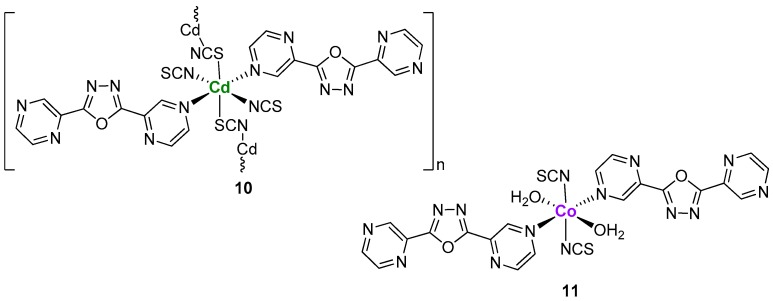
Structures of the Cd(II) and Co(II) complexes with ligand 2,5-bis(pyrazine)-1,3,4-oxadiazole (Lc).

X-ray structural analysis of **10** revealed a 1-D coordination array where Cd(II) shows an octahedral coordination geometry with Lc acting as monodentate ligand in the cisoid-I conformation (differently from what happened in the case of silver ions) (Figure 5). The X-ray structure of complex **11,** instead, showed the central Co(II) atom coordinated to two Lc ligands in a monodentate fashion, two nitrogen atoms from SCN and two H_2_O molecules (Figure 5), while the crystal packing of this molecule showed a 3-D network generated from multiple hydrogen bonding interactions involving all the ligands coordinated to Co(II). In this paper, the author stressed once again the critical role of the metal center in the final structure. In particular, while the Cd(II) and the Co(II) centers coordinated similarly the oxadiazole-based ligand Lc, they showed differences in the coordination of the ancillary ligand thyocianate. As expected from the hard–soft acid–base concepts, Cd(II) coordinated to both N and S atoms of SCN while Co(II) only preferred the N-terminal [13].

**Figure 6:**
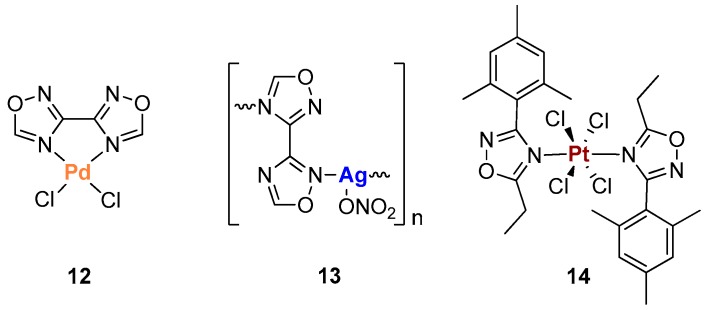
Structures of metal complexes with ligand bearing the 1,2,4-oxadiazole scaffold.

1,2,4-Oxadiazoles have been, by far, less explored as ligands for metal complexes compared to 1,3,4-oxadiazoles. In 2007, the group of Steel published a paper with the indicative title “The first metal complexes of 3,3′-bi-1,2,4-oxadiazole: A curiously ignored ligand” [18]. The authors stressed the multi-chelating potential of this bi-1,2,4-oxadiazole ligand and synthesized a mononuclear palladium(II) complex (**12**) and a one-dimensional silver(I) coordination polymer (**13**) (Figure 6). Compound **12** was obtained by reacting the bi-1,2,4-oxadiazole ligand with Li_2_PdCl_4_ under neutral conditions, while when the same reaction was performed with PdCl_2_ in 2 M HCl solution a ring-opened hydrolysis product was obtained. Finally, the authors explored also the possibility of obtaining metallo-supramolecular assemblies by using Ag(I) nitrate as metal salt. In the coordination polymer **13,** the ligand acts in a bridging mode (Figure 6), while the nitrate anions oscillate with the polymer itself in a wave-like fashion. In both compounds **12** and **13**, the ligand coordinates the metal through the N4 nitrogens and the authors proposed the greater basicity of this position compared to the N2 atoms as possible explication [18]. 

Even though Steel published the first metal complex with bi-1,2,4-oxadiazole, four years before, in 2003, Bokach et al. reported, to the best of our knowledge, the first metal complex containing a 1,2,4-oxadizole ring so far [19]. The main aim of the study of Bokach and collaborators, nevertheless, was not to synthesize a metal complex, but rather to develop a general route for the synthesis of 1,2,4-oxadiazoles based on the [2 + 3] cycloaddition between metal-activated nitriles and nitrile oxides under mild conditions. The authors performed reactions at room temperature and in CH_2_Cl_2_ of Pt(IV) complex [PtCl_4_(CH_3_CH_2_CN)_2_] with stable aryl nitrile oxides, i.e., 2,4,6-Me_3_C_6_H_2_CNO. After one day the final 1,2,4-oxadiazole Pt(IV) complex **14** was obtained in good yields. Finally, in order to produce the liberation of the free heterocycle, the platinum(IV) complex was treated with an excess of pyridine in chloroform, giving free 1,2,4-oxadiazoles in solution and a precipitate of the complex trans-[PtCl_4_-(pyridine)_2_]. The authors evaluated also the possibility of obtaining the release of the free 1,2,4-oxadiazole ligand from the corresponding Pt(II) compound but without success [19]. 

## 3. Biological Applications

**Figure 7:**
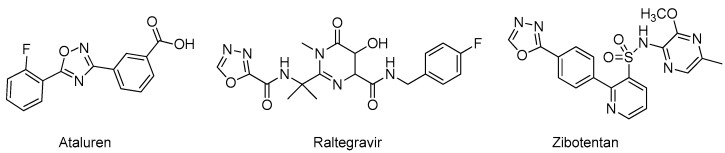
Structures of oxadiazole-based molecules of great interest in medicinal chemistry.

Medicinal chemistry is the field where oxadiazole-containing molecules had the greatest impact. Many oxadiazole-based molecules have been developed against cancer, inflammation, infection, diabetes, and other diseases [1,4,5,8]. The compounds ataluren and zibotentan (Figure 7) are in late-stage clinical trials for the treatment of cystic fibrosis and prostate cancer, respectively [20,21]. Remarkably, the compound raltegravir made it into the market for the treatment of HIV infection [22]. 

Considering the large amount of literature concerning the biological activity of oxadiazole-based compounds, is somehow surprising that only few examples of biologically active metal complexes containing oxadiazole ligands have been reported so far.

**Figure 8:**
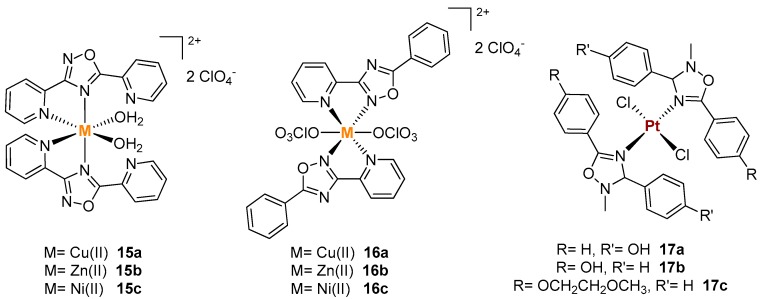
Structures of Ni(II), Cu(II), and Zn(II) complexes with ligands 3,5-bis(2′-pyridyl)-1,2,4-oxadiazole and 3-(2′-pyridyl)5-(phenyl)-1,2,4-oxadiazole, respectively.

Between 2010 and 2011, Terenzi et al. described the synthesis of Ni(II), Cu(II), and Zn(II) complexes of two chelating 1,2,4-oxadiazole ligands, namely 3,5-bis(2′-pyridyl)-1,2,4-oxadiazole and 3-(2′-pyridyl)5-(phenyl)-1,2,4-oxadiazole, obtaining the series **15a-c** and **16a-c** depicted in Figure 8 [23,24]. The authors decided to focus their attention on the anticancer activity of the Cu(II) derivative **15a [23]**. In particular, the impact of the ligand and of its metal complex on human hepatoblastoma HepG2 and colorectal carcinoma HT29 cell lines was evaluated and correlated to their potential DNA binding activity. While the ligand resulted to be widely inactive, compound **15a** reduced the vitality of both cell lines, displaying IC_50_ values around 10 µM after 24 h of treatment. Furthermore, cell morphological and flow cytometry analyses revealed an apoptotic death induction of the cancer cells with DNA fragmentation confined in the sub-G0/G1 phase of cell cycle. UV-Vis and circular dichroism titrations of **15a** with calf thymus DNA (ct-DNA) indicated a tight binding of the copper complex to the B-DNA model (binding constant K_b_ equal to 2.2 × 10^4^ M^-1^) and the authors interpreted the different spectroscopic changes as indicative of a groove binding mode without intercalation. The latter hypothesis was confirmed by viscosity and gel electrophoresis experiments in which **15a** did not modify the relative viscosity of DNA and, at the same time, did not degrade the polynucleotide nor affected its mobility [23]. 

**Figure 9:**
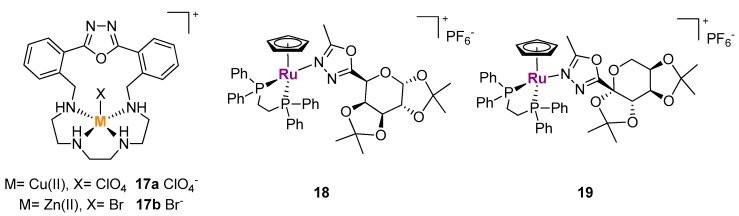
Structures of metal complexes with 1,3,4-oxadiazole ligands with biological activity.

In doing their work about the potential anticancer activity of metal-based 1,2,4-oxadizole compounds, Terenzi and colleagues were inspired by a previous study performed in the laboratory of Wagner in 2008, when a series of platinum(II) complexes bearing ∆^4^-1,2,4-oxadiazoline ligands (saturated oxadiazoles) have been successfully tested against human ovarian, colon, and testicular cancer cell lines [21]. The synthesized neutral trans-platinum(II) derivatives **17a** and **17b** (Figure 9) were relatively active also in cisplatin resistant cell models and the authors suggested that the heterocyclic ligands could allow for additional interactions compared to cisplatin. For instance, it was rationalized that the substituted phenyl groups attached to the five-membered ring could intercalate into the DNA, preventing repair mechanisms by the Pt-resistant cells [21].

In 2012, Terenzi et al. decided to extend their chemical-biology studies to metal complexes bearing 1,3,4-oxadiazole ligands. In particular, the Cu(II) and Zn(II) complexes **17a** and **17b** (Figure 9), containing an oxadiazole macrocyclic ligand, have been tested for their cytotoxic activity toward human breast carcinoma cells and for their interaction with DNA in solution [25]. Compounds **17a** and **17b** have been synthesized and characterized in solution previously by Ambrosi et al. (see “Metal ion sensing” section below) and in that study it was demonstrated that the apical ligand of both complexes is replaced by a H_2_O molecule providing a dicationic species at neutral pH [26]. The cationic nature of the complexes (translating in good solubility in physiological conditions), together with the presence of the potential DNA-intercalating 2,5-diphenyl[1,3,4]oxadiazole planar moiety, were considered promising properties by Terenzi and collaborators who performed several spectroscopic interaction studies with ct-DNA. Both the Cu(II) and Zn(II) complexes distinctly increased the melting temperature of the B-DNA model and markedly modified its circular dichroism spectrum indicating a strong interaction between **17a–b** and the double helix. Ethidium bromide displacement assays revealed an intercalative mode of binding of the complexes to DNA, with the copper derivative showing higher affinity than the zinc one. As a further DNA binding mode, the authors suggested the possibility that the apical position of the complexes could be replaced by a nucleophilic position of a DNA base, even if this hypothesis requires additional studies. Human breast carcinoma MDA-MB-231 cells exposed to **17a** and **17b** did not show any substantial change in their growth rate or cell cycle profile. This effect was attributed by the author to a scarce uptake of the metal complexes by the cells. When cell viability experiments were repeated in the presence of a lipidic carrier (Lipofectamine 2000) the cell survival was reduced up to about 58 and 31% with **17a** and **17b**, respectively. The negative correlation with the cell-free spectroscopic studies suggested that DNA binding, is, probably, not the only mechanism of the cytotoxicity induced by the metal complexes [25].

Recently, the group of Fernandes and co-workers reported on the synthesis and cytotoxicity of new η^5^-cyclopentadienyl Ru(II) complexes with galactose and fructose carbohydrate derivative ligands functionalized with 1,3,4-oxadiazole N-coordinating moieties, compounds **18** and **19** respectively (Figure 9) [27]. Both compounds displayed good IC_50_ values (around 5 µM) against human cervical carcinoma cells (HeLa) after 48 h of continuous exposure. Unfortunately, besides performing the MTT assay, the authors did not suggest any mechanism for the activity of their compounds [27].

## 4. Applications in Optoelectronic Materials

Oxadiazole heterocyclic derivatives are characterized by very interesting luminescent behaviors, electron mobility, and very good thermal stability. These qualities make them good candidates for the construction of new organic/hybrid materials (molecular or polymeric) for applications as optoelectronic devices (e.g., organic solar cells [28,29]), sensors for various metal-ions (vide infra), and also as liquid crystals [30,31]. Among these applications, oxadiazoles were largely employed in the preparation of organic light-emitting diodes (OLED). The reason for their success is due to fact that an oxadiazole ring is electron deficient, and it is able to block the movement of holes but also able to conduct electrons well.

In the last 20 years, several examples of electroluminescent devices employed oxadiazole derivatives in both molecular- [32,33,34,35] and polymer-based [36,37,38,39,40,41] LEDs. In many examples, the oxadiazole unit was used either as electroconducting material or as emitting species. Concerning the first type of application, in the early examples, oxadiazoles were blended with numerous electroluminescent polymers [42,43]. However, in some cases, incompatibilities with such polymers occurred that led to phase separation or crystallization, especially at higher temperatures. In order to avoid such problems, a different approach was used to improve the charge-transporting properties in oxadiazoles-based OLEDs. Polymers were synthesized with oxadiazole units placed in either the main or the side chain. The use of polymeric species allows also simpler and milder fabrication conditions (e.g., spin-coating and ink-jet printing) for the preparation of thin films compared to small organic molecules that are usually assembled via vacuum depositions. For this reason, a great deal of attention was focused on oxadiazole-based polymers.

A good example of simple and cost-effective design of OLED was provided by Zhang et al. [38], where polycarbazoles with oxadiazole pendants were synthesized by the Suzuki coupling reaction. The copolymers exhibited blue emission with significantly improved fluorescence quantum efficiencies compared to their analogous polymers without oxadiazole units. The presented polymers were found to perform well as electron transport hosts for one of the frequently employed red-emitters Ir(ppq)_2_(acac) (where ppq is bis-2,4-diphenylquinolinato and acac is acetylacetonate) in red OLEDs, due to good spectral matching with the absorption spectra of the iridium complex.

**Figure 10:**
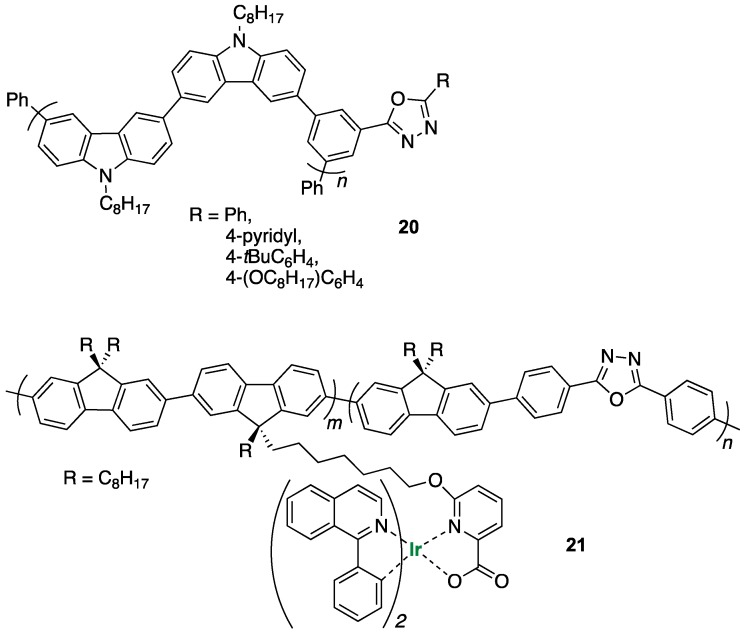
Structures of reported polymeric structures bearing 1,3,4-oxadiazole units.

Using a similar synthetic approach Monkman and co-workers [40] also presented the synthesis and a comprehensive study of the photophysical properties of a series of bicarbazolyl–oxadiazole oligomers (**20** in Figure 10) and established their suitability to host a green phosphorescent emitter (Ir complex) in OLEDs using a simple device architecture constructed by solution processing.

Deng et al. reported an interesting synthetic strategy to improve the electroluminescence (EL) in polymeric OLEDs [39]. They prepared a series of the fluorene-alt-oxadiazole phosphorescent copolymers containing the pendent iridium(III) complex unit (**21** in Figure 10). All these **21**-based OLEDs exhibited identical saturated red emissions with a maximum peak at around 628–634 nm and the best device performance showed a maximum luminance of 1125 cd/m^2^ at 12 V and a maximum current efficiency of 1.2 cd/A at 63 mA/cm^2^. This demonstrated that incorporation of the oxadiazole unit into the phosphorescent copolymers and the direct connection of the emitting Ir complex have positively influenced the EL performance of this OLED.

Metal complexes bearing oxadiazole ligands were used as emitting species in light-emitting diodes. Introducing different functionalized oxadiazole ligands allows the emission color to be easily tailored, thermal stability, and film formation properties of such complexes.

**Figure 11:**
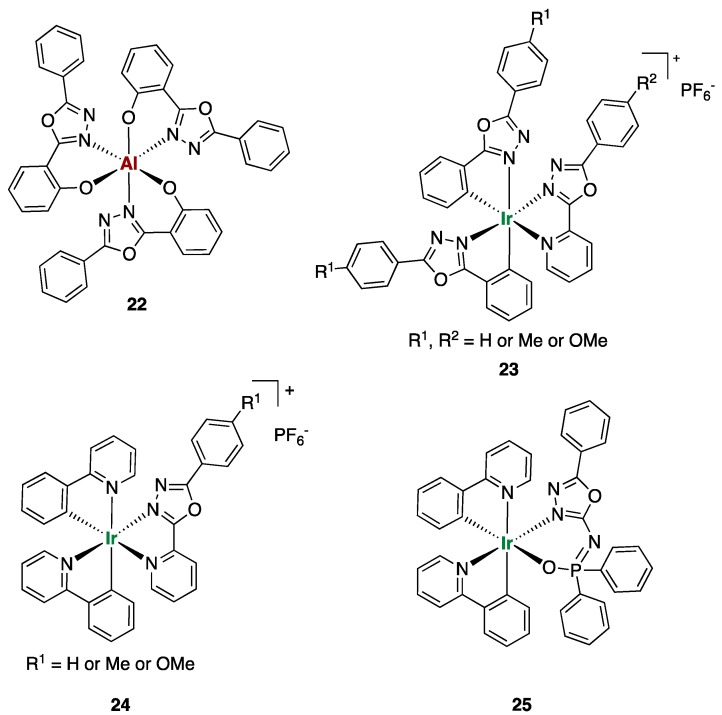
Structures of reported Al(III) and Ir(III) complexes bearing 1,3,4-oxadiazole ligands employed as emitters in organic light-emitting diodes (OLEDs).

Kippelen and co-workers showed in 2009 an interesting example of bright blue emitting OLEDs based on an aluminum complex characterized by a 2-(5-phenyl-1,3,4-oxadiazol-yl)phenonate ligand (**22** in Figure 11) [32]. The authors showed how **22** is a better material for OLEDs than the well-established organic electron transporter 2-(4-biphenylyl)-5-(4-*tert*-butylphenyl)-1,3,4-oxadiazole (PBD) [43] and a good electron transporter comparable with tris(8-hydroxyquinoline)aluminum (Alq3). These results gave a considerable boost to the search for alternative oxadiazole complexes to be employed as emitters for OLEDs. An example of such a pursuit is displayed by the work of by Feng et al. [33]. Ligands formed by 8-hydroxyquinoline connected to oxadiazole moieties were employed in the preparation of novel aluminum complexes. The full photophysical characterization of such complexes showed the great potential of this material for emitting blue light [33].

Changing from aluminum to iridium complexes allowed the preparation of OLEDs of different color emissions other than blue. Bryce and co-workers reported the synthesis and the optoelectronic characterization of new ionic iridium(III) complexes (**23** and **24** Figure 11) [34]. Complexes **23** and **24** are all characterized by 2,5-diaryl-1,3,4-oxadiazole ligands functionalized with different substituents. Changing the substituents on the oxadiazole ligands (**23**, Figure 11) or replacing two oxadiazole ligands with phenylpyridine (**24**, Figure 11) changed the photo- and the electroluminescence properties of the light-emitting electrochemical cells (LECs) prepared. Complex **23** is a yellow emitter (λ_max_, EL 552–564 nm) whereas complex **24** is a red emitter (λ_max_, EL 616–626 nm). A yellow LEC with maximum brightness of 3125 cd/m^2^ at 14 V has been achieved using **23** (R_1_, R_2_ = H) as the emitter. On the other hand, the red-emitting LEC prepared with **24** (R_2_ = H) displayed very high efficiency and very high brightness (8528 cd/m^2^, at 10 V).

Zhai and collaborators utilized similar design for their Ir(III) complexes, except for the insertion in the oxadiazole ligand of amide substituents (bezamide or diphenylphospinic amide) that acted as ancillary ligands [35]. The incorporation of the phosphine oxide group (**25**, Figure 11) into the molecule further enhanced the electron mobility of the complex due to the two electron transport units: phosphine oxide and 1,3,4-oxadiazole. The phosphorescent green OLED prepared showed good performances with current efficiency for devices remaining over 60.6 cd/A even at a brightness of 10,000 cd/m^2^. The research work presented over the last two decades showed how largely oxadiazole derivatives were employed in different types of OLEDs and how this research topic is still prolific.

## 5. Metal Ion Sensing

The development of optical molecular sensors for the detection of a specific analyte is a growing area of chemistry. The appeal of molecular sensors is due to the fact that they offer many advantages in terms of sensitivity, response time, and costs with respect to other detection methods, such as the expensive and time-consuming inductively coupled plasma–mass spectroscopy and atomic absorption spectroscopy [44]. Among the possible substrates, metal ions have a central role since they are almost ubiquitous in the functions governing life. Therefore, their selective detection and quantification raises considerable attention in many fields such as environmental and security monitoring, waste management, nutrition, and clinical toxicology [45].

In this context, oxadiazoles have been shown to be an optimal scaffold to build optical sensors due to their characteristics. In particular, oxadiazoles have excellent photophysical properties, high chemical stability, and they can be easily functionalized with different chromophores and chelating groups. The latter enable the employment of two different strategies: one where the oxadiazole unit is connected to a receptor that upon binding the analyte (metal ion) alters its electronic communication with the oxadiazole unit, creating a detectable signal, and another strategy, where the oxadiazole chromophore is part of the receptor unit and, upon the binding event, photophysical perturbations of the chromo- or the fluorophores occurs. A variety of photophysical mechanisms are employed in governing the sensing phenomena including photoinduced electron transfer (PET), excimer/exciplex formation, photo-induced energy transfer, intramolecular charge transfer (ICT), and excited state intramolecular proton transfer (ESIPT). For example, PET systems generally produce signaling via fluorescence enhancement or quenching (‘ON–OFF’ or ‘OFF–ON’ states), meanwhile ICT probes could offer detection by modulation of both absorption and emission properties.

**Figure 12:**
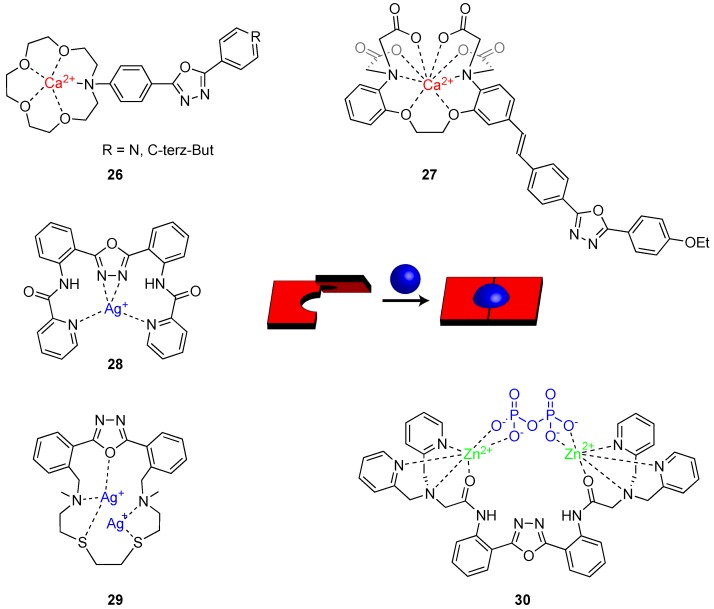
Structures of reported metal ion sensors bearing 1,3,4-oxadiazole units.

Mashraqui et al. presented efficient chemoinophores **26** (Figure 12) where a N-phenylaza-15-crown-5 ether was connected to an aryl/heteroaryl oxadiazole to function as the new intramolecular charge transfer (ICT) probes [46]. The authors presented a detailed study of the photophysical properties of this probe in the presence of selected metal ions including Ca^2+^, Ba^2+^, Mg^2+^, Na^+^, K^+^, and Li^+^. The ICT bands in both UV–Vis and emission spectra experienced varying degrees of blue shifts based on the different cation affinities (binding strength: Ca^2+^ > Ba^2+^ >> Li^+^ > Na^+^ > K^+^ > Mg^2+^). The blue shift is caused by the removal of the aza-crown ether nitrogen from the conjugated system of the oxadiazole. Titration experiments performed in a matrix of ions also indicated superior interaction of **26** with Ca^2+^, as demonstrated by their relatively high binding interaction for Ca^2+^ (logK_s_ = 3.55–3.10) compared to the biologically interfering Mg^2+^ (logK_s_ = 1.67–1.30) and alkali metal ions (log K_s_ = 0.54–2.14).

Another fluorescent probe for Ca^2+^ based on a 1,3,4-oxadiazole derivative has been reported by Choi and co-workers [47]. Their molecular design strategy involved the incorporation of 2-(4-ethoxyphenyl)-5-(4-methylphenyl)-1,3,4-oxadiazole as the fluorophore and 1,2-bis(2-aminophenoxy)-ethane-N,N,N′,N′-tetraacetic acid (BAPTA) group as the Ca^2+^ recognition site (molecule **27**, Figure 12). The electron-deficient oxadiazole unit was conjugated to the electron-rich groups of ethoxybenzene and the salt form of BAPTA, leading to the formation of a push–pull conjugated molecule characterized by an ICT along its large π-electron conjugation system. In addition, since there was an overlap of the excitation spectrum of the oxadiazole (260–360 nm) with that of BAPTA (230–320 nm), the same excitation wavelength could be employed to trigger the emission of both the oxadiazole fluorophore and BAPTA. Once BAPTA bound a Ca^2+^ ion, its electron-donating properties are restricted with a concomitant reduction of π-electron conjugation of the entire probe. This results in a blue shift of both absorption and emission spectra. The probe **27** displayed high sensitivity and selectivity for Ca^2+^ over other metal ions, a large Stokes shift of 202 nm, and enabled radiometric emission measurement with an obvious color change.

The later characteristics, together with ease of cell-membrane permeability, allowed the authors to use **27** to monitor in situ the different the intracellular [Ca^2+^] by confocal microscopic imaging. Human umbilical vein endothelial cells (HUVEC) were loaded with **27** and confocal fluorescent imaging was performed at an excitation wavelength of 458 nm. The results presented clearly indicated the capability of **27** to determine the intracellular [Ca^2+^] in real-time after the living cells were exposed to the drug. A year later, the same authors employed a similar strategy for the preparation of a Cd^2+^ fluorescent probe [48]. In this case the molecule was characterized by a BAPTA receptor and a two oxadiazole units. The probe exhibited high selectivity for Cd^2+^ and a low detection limit of 20 nM in aqueous solution, making it useful for Cd^2+^ imaging in living MCF-7 cells.

Oxadiazoles also proved to be efficient photoinduced electron transfer (PET)-type ion sensors. These ion probes are devised to covalently link fluorophores with a receptor by means of non-conjugating spacer groups and reversibly switch fluorescent intensity ON, after binding the desired ion. Zheng et al. reported an interesting example of a PET ion sensor [49], where they employed 1,3,4-oxadiazole as the fluorophore and connected it to two pyridine-2-formamidophenyl (**28**, Figure 12). The nitrogen atoms of pyridine rings are both the cation receptor and the quenching agent that operate via PET. Recognition of the metal ion sequestered the lone pairs in nitrogen atoms and rebuilt a rigid (planar) molecular system (see sketch in Figure 12), which stopped the PET quenching and produced a fluorescent enhancement in the 1,3,4-oxadiazole emission. Sensor **28** showed a remarkable enhancement (342%) in fluorescence after addition of Ag^+^.

Several assays demonstrated that this probe was specifically selective and sensitive for Ag^+^ over competing cations (Al^3+^, Ba^2+^, Cd^2+^, Cr^3+^, Fe^2+^, Fe^3+^, Hg^2+^, K^+^, Mg^2+^, Mn^2+^, Na^+^, Pb^2+^, and Zn^2+^) in HEPES buffer solution. During recognition, a stable 1:1 **28**-Ag^+^ complex formed (Figure 12). Usually, highly selective probes for transition metal ions that give a positive response rather than fluorescent quenching are not so easy to obtain. Therefore, the design of Zheng et al. demonstrated how **28** could be a viable candidate for fluorescent sensing of Ag(I) ions.

Another example of a PET-regulated oxadiazole probe was reported by Fusi and co-workers, who described the coordination behavior toward Cu(II), Zn(II), Cd(II), and Pb(II) of a series of macrocyclic fluorescent sensors [26]. Three polyaza macrocycles were prepared with different cavity sizes and numbers of secondary amine functions, but in each of them a 2,5-diphenyl[1,3,4]oxadiazole unit was inserted (only the tetraaza macrocycle **17b** is presented in Figure 9). All the ligands were highly soluble in water in the examined range of pH 2–12, and their photochemical properties were PET regulated, as the ligands were highly fluorescent in strongly acidic media and quenched beginning from about pH = 8. Among the three ligands prepared, only **17b** showed the optimal cavity size and the appropriate number of nitrogen donor groups to selectively bind Zn^2+^ ions. The oxadiazole-based ligand in compound **17b** behaves as an efficient OFF–ON sensor for Zn^2+^ at physiological pH, even in the presence of interfering species such as Cd^2+^ and Pb^2+^, since its geometry consents to the full participation of all of the donor atoms in the metal coordination of Zn^2+^. This prevents the PET from giving rise to a significant enhancement of fluorescence, that can be seen even with the naked eye.

In a following publication [50], the same authors in collaboration with Prodi’s group reported a macrocycle similar to the one in **17b** where two thioether groups together with two tertiary amine functions were inserted (see **29** in Figure 12). The aim of this modification was to make the new ligand **29** able to coordinate both soft and heavy metal ions. In acetonitrile solution, the fluorescence of the chemosensor changed upon addition of different metal ions, such as Cu^2+^, Zn^2+^, Cd^2+^, Pb^2+^, Hg^2+^, and Ag^+^. NMR experiments pointed out that a not fluorescent ML species and a fluorescent M_2_L species characterized the binding mode of **29**. The inclusion of **29** inside core-shell silica nanoparticles (PluS-NP) led to high water solubility, allowing the performance of the metal detection without the use of additional solvents. Moreover, the interaction of the chemosensor with the NP induced also other advantages such as a higher selectivity. In fact, while in CH_3_CN **29** was able to complex a large number of cations, **29**@PluS-NP was responsive only to the presence of Ag^+^ and Hg^2+^. Finally, due to the high degree of loading reachable in the PluS-NPs, the authors demonstrated how to control the stoichiometry of the formed complex upon changing the number of ligands per nanoparticle. This possibility can be used as an additional tool for the tuning of the affinity and selectivity of these types of sensing systems.

Among the different types of fluorescent ion sensors, it is worth mentioning the ones characterized by the excited state intramolecular proton transfer (ESIPT) signaling mechanism. Fluorescent sensors with ESIPT property are very attractive due to the two wavelength emissions and large Stokes shift. In 2015, Tang et al. designed a new sensor constituted by a 2,5-diphenyl[1,3,4]oxadiazole connected to two dipicolyl amine (**30**, Figure 12) [51]. Ligand **30** showed highly selective and ratiometric fluorescence responses to Zn^2+^ in a 1:2 binding ratio. Fluorescence spectra and ^1^H-NMR studies demonstrate that **30** binds Zn^2+^ ions through its amide form, which promoted the ESIPT emission enhancement. The in situ generated **30**–2Zn^2+^ complex was further used as a recognition sensor for pyrophosphate (PPi) (via further complexation as depicted in Figure 12). Instead, if sulphide S^2-^ anions were present in solution, the Zn^2+^ in **30**–2Zn^2+^ is sequestered with the consequent release of free ligand **30**. The authors used this multi-analyte recognition of **30** to build an INHIBIT logic gate based on the fluorescence intensity of **30**–2Zn^2+^ at 396 nm. Depending on the two chemical inputs, namely, input 1 (PPi) and input 2 (S^2-^), **30**–2Zn^2+^ can switch between high and low emission states. Once a threshold value is set at max of emission (396 nm), the output is recorded as 1 and 0 corresponding to the strong and weak emission intensity respectively. Input 1 elicits strong emission at 396 nm, represented as output ‘1′. On the contrary, input 2 results in a weak emission signal, represented as output ‘0′. Therefore, monitoring the emission intensity at 396 nm, upon addition of PPi and S^2-^, and their mixture results in an INHIBIT logic gate. In this regard, it is worth mentioning the report form Jiang and co-workers where they prepare different molecular logic gates and switches based on 1,3,4-oxadiazoles triggered by metal ions [52].

In conclusion, oxadiazoles were demonstrated to be great building blocks for the construction of different types of ion sensors. Their interesting photophysical properties and chemical tunability are remarkable and allow them to be paired with several receptor units. This enables their employment in many strategies for signaling the recognition moment using different photophysical mechanisms as shown in the above-mentioned examples.

## 6. Conclusions

In this review article we highlighted how oxadiazole ligands are ideal scaffolds for creating new coordination compounds with great potential for different fields. While there is a vast literature on metals interacting with oxadiazoles to afford new materials, studies on the biological activity of metal complexes involving oxadiazole ligands are by far limited. This is quite curious considering that oxadiazole derivatives have been known for ages for their wide spectrum of biological activities including anticancer, antibacterial, and anti-inflammatory [1,8]. We urge the medicinal inorganic chemistry community to take advantage of the multiple properties of this precious class of ligands. New metal complexes of oxadiazoles could find, for example, huge applications as anticancer drug candidates. There is, in fact, a big need in cancer chemotherapy (and immunotherapy) to find new metal compounds with improved efficacy and less side effects than the clinically used platinum drugs [53]. New complexes combining the properties of platinum and the ones of known oxadiazole ligands, for example, could serve the scope. Another interesting approach could be to couple in a single complex the promising anti-inflammatory and anticancer properties of oxadiazoles and gold derivatives [54]. Besides, the photochemical behavior of oxadiazoles (in particular 1,2,4-oxadiazoles) has been widely investigated [3,6] and could be implemented for the preparation of new photoactivatable Pt(IV) prodrugs which, in recent years, have witnessed a remarkable increase of attention in cancer research [55].
